# Chronic aerobic swimming exercise promotes functional and morphological changes in rat ileum

**DOI:** 10.1042/BSR20150001

**Published:** 2015-10-19

**Authors:** Layanne Cabral da Cunha Araujo, Iara Leão Luna de Souza, Luiz Henrique César Vasconcelos, Aline de Freitas Brito, Fernando Ramos Queiroga, Alexandre Sérgio Silva, Patrícia Mirella da Silva, Fabiana de Andrade Cavalcante, Bagnólia Araújo da Silva

**Affiliations:** * Programa de Pós-graduação em Biologia Celular e Molecular/Centro de Ciências Exatas e da Natureza/Universidade Federal da Paraíba, Brazil, 58051-090; † Programa de Pós-graduação em Produtos Naturais e Sintéticos Bioativos/Centro de Ciências da Saúde/Universidade Federal da Paraíba, Brazil, 58051-970; ‡ Departamento de Educação Física/Centro de Ciências da Saúde/Universidade Federal da Paraíba, Brazil, 58051-900; § Departamento de Biologia Molecular/Centro de Ciências Exatas e da Natureza/Universidade Federal da Paraíba, Brazil, 58051-090; ║ Departamento de Fisiologia e Patologia/Centro de Ciências da Saúde/Universidade Federal da Paraíba, Brazil, 58051-900; ¶ Departamento de Ciências Farmacêuticas/Centro de Ciências da Saúde/Universidade Federal da Paraíba, Brazil, 58051-900

**Keywords:** aerobic exercise, contractile reactivity, gastrointestinal tract, oxidative stress, rat ileum, swimming

## Abstract

Several studies have reported the gastrointestinal (GI) effects promoted by the physical exercise. Thus, we aimed to evaluate the influence of swimming exercise on the contractile reactivity, lipid peroxidation and morphology of rat ileum. Wistar rats were divided into sedentary (SED) and groups exercised for two (EX2), four (EX4), six (EX6) or eight (EX8) weeks, 5 days/week. Animals were killed; the ileum was removed and suspended in organ baths where the isotonic contractions were recorded. Lipid peroxidation was evaluated by MDA (malondialdehyde) measurement with TBARS (thiobarbituric acid reactive substances) assay and morphology by histological staining. Cumulative concentration-response curves to KCl were attenuated, as the *E*_max_ values were changed from 100% (SED) to 63.1±3.9 (EX2), 48.8±3.8 (EX4), 19.4±1.8 (EX6) and 59.4±2.8% (EX8). Similarly, cumulative concentration-response curves to carbamylcholine hydrochloride (CCh) were attenuated, as the *E*_max_ values were changed from 100% (SED) to 74.1±5.4 (EX2), 75.9±5.2 (EX4) and 62.9±4.6 (EX6), but not in the EX8 (89.7±3.4%). However, CCh potency was increased in this latter, as the EC_50_ was altered from 1.0±0.1×10^−6^ (SED) to 2.1±0.4×10^−7^ (EX8). MDA concentration was altered only in EX4 (44.3±4.4) compared with SED (20.6±3.6 μmol/l). Circular layer was reduced in SED when compared with the exercised groups. Conversely, longitudinal layer was increased. In conclusion, chronic swimming exercise reduces the ileum contraction, equilibrates the oxidative damage and promotes changes in tissue size to establish an adaptation to the exercise.

## Abstract (precis)

In the present study, we evaluated the influence of chronic swimming aerobic exercise on the contractile reactivity, lipid peroxidation and tissue morphology of small intestine from rats. We showed that, accordingly to the time of exercise, the rat ileum has its contractile reactivity reduced, in addition to the increased lipid peroxidation followed by its reestablishment to basal levels, as well as the augmented longitudinal smooth muscle layer thickness together with the reduced circular thickness.

## INTRODUCTION

Exercise is an activity that affects all organs and tissues and can result in many health benefits [1], such as decreasing the vasoconstriction resulting from aging [2] and through changes on cardiovascular system in order to maintain tissue oxygen demand [3–5]. It has been considered an important therapeutic tool in the prevention and treatment of various diseases such as cardiovascular, metabolic syndrome and gastrointestinal (GI) disorders [6–8].

The exercise promotes positives effects on GI tract such as reduced incidence of colorectal carcinoma, diverticulitis, cholelithiasis and constipation. Despite this, strenuous exercise may cause GI symptoms, such as nausea, vomiting, diarrhoea and intestinal bleeding. Thus, recommendations to reduce exercise-induced GI-symptoms include reduction in exercise intensity and prevention of dehydration [7,9].

It is well described that exercise is responsible for stimulating the reactive oxygen species (ROS) and reactive nitrogen species (RNS) production during muscle contraction and it plays an important role in muscle metabolism. Studies have reported that in a physiological range that is low to moderate levels, ROS provide several regulatory functions in the cell, such as the control of gene expression, regulation of signalling pathways and modulation of skeletal muscle contraction force, leading to an increase on muscle strength [10]. However, increased ROS and RNS concentrations promote changes in the lipid matrix and cell membranes, characterizing the oxidative stress [10–13]. In this context, the long-term exercise, namely chronic exercise, in moderate intensity, promotes increase in antioxidant defences, which remove free radicals (FRs) and stabilize the reactive species production [14].

Although intestinal smooth muscle is not directly involved in physical exercises, it is subjected to physiological stress, especially for the ischaemia-reperfusion process due to the diversion of blood flow to the skin and the active skeletal muscles [15]. This ischaemia can promote motor and intestinal mucosa changes [16]. The effect of exercise has been extensively studied on the intestinal mucosal layer, absorption of nutrients and GI permeability [17,18]. However, few studies have reported the effect of exercise on intestinal contractile response, despite the smooth muscle reactivity abnormalities represent one of the pathophysiological processes that characterize intestinal colic, diarrhoea and constipation [19].

Even though studies have evaluated the reactivity of ileum using the treadmill exercise, the physiological responses of treadmill exercise differs from swimming exercise, as well as the use of swimming exercise shows advantages over the treadmill protocol, because swimming is a natural ability of rats; therefore, is widely used as an appropriate model of physical exercise, whereas to running, rats must be conditioned, does not representing a natural instinct [20,21]. However, there are many works relating the effects of treadmill in physiological parameters instead swimming exercise, whereas it is a common human-sport practice.

Therefore, in the present work, we investigated the influence of chronic swimming aerobic exercise on the contractile reactivity of intestinal smooth muscle, oxidative stress and morphology of rat ileum hypothesizing that the regular practice of swimming exercise in a moderate intensity can provide beneficial effects for the GI system.

## MATERIALS AND METHODS

### Ethical approval

All experimental procedures were performed following the principles of animal care of the Guidelines for the ethical use of animals in applied aetiology studies [22] and previously approved by UFPB Ethics Committee on Animal Use (Protocol/CEUA no. 0907/13).

### Animals

Wistar rats (*Rattus norvegicus*), initially 2-months-old, weighing 180–200 g, were obtained from the bioterium of the Biotechnology Center (CBiotec)/UFPB. The animals were kept under restricted food control with balanced diet (Labina®), to avoid large differences in body weight and density and had access to water *ad libitum*. They were maintained in rooms at 21±1°C and submitted to a 12 h light-dark cycle (light from 6 to 18 h). Forty-eight hours after the last exercise session, the animals were fasted for 18 h (receiving only water *ad libitum* during this period) and were then killed by cervical dislocation followed by cervical vessels section to perform the experimental analysis. This time of fasting is important to avoid the influence of substances released by the GI tract during the intestinal transit.

### Drugs

Calcium chloride bihydrate (CaCl_2_.2H_2_O), magnesium chloride hexahydrate (MgCl_2_.6H_2_O) and glucose (C_6_H_12_O_6_) were purchased from Vetec. Sodium bicarbonate (NaHCO_3_) was purchased from Fmaia. Sodium chloride (NaCl) and potassium chloride (KCl) were purchased from Química Moderna. Monopotassium phosphate (NaH_2_PO_4_), sodium hydroxide (NaOH) and hydrochloric acid (HCl) were purchased from Nuclear. Carbamylcholine hydrochloride (CCh) was purchased from Merck. Formaldehyde was purchased from Vetec. Thiobarbituric acid, tetramethoxypropane, perchloric acid, Mayer's haematoxylin and eosin were purchased from Sigma. Carbogen mixture (95% O_2_ and 5% CO_2_) was obtained from White Martins (Brazil).

The CCh was used to mimic the cholinergic stimulation that happens on the intestinal smooth muscle promoted by the myenteric plexus [23,24]. The KCl was employed to simulate the pacemaker of interstitial cells of Cajal located at the boundaries and in the substance of the inner, circular muscle layer, from which they spread to the outer, longitudinal muscle layer [25,26].

### Exercise protocol

In swimming protocols for rats, the animals swim vertically and are submitted to exercise with overloads tied to the thorax [27–30]. Brito et al. [31] showed that rats submitted to forced swimming exercise for 1 h with a metal ring of 3%-6% of theirs body weight attached to their torso present blood lactate levels into the range of aerobic exercise, characterizing a moderate intensity. Thus, based on these evidences, we performed the exercise protocol with rats into a restricted range of age with a metal of 3% of their body weight attached to their body to avoid the inherent ability of rats to remain floating on the water surface.

The animals were divided into five groups (five animals each): sedentary (SED) and exercised for two (EX2), four (EX4), six (EX6) and eight weeks (EX8). Before the experiments, animals were placed in a container with 1.5 cm of water at 23°C-25°C for 2 min, for the animal acclimation. This procedure was important to prevent the animal stress, especially on start of the exercise [32]. The swimming protocol was adapted from Chies et al. [33] and was performed in a plastic container measuring 43×63×33 cm, with water at a temperature of 23°C-25°C. The animals of SED group (control) were subjected to the same stress of exercised groups, including food deprivation and exposure to noise throughout the training. Exercised animals were subjected to daily forced swimming for 1 h, 5 days per week, between 8 a.m. and 4 p.m., kept attached to a metal ring corresponding to 3% of their body weight on its trunk, which improves the resistance of the animal to exercise and prevents the fluctuation [22,27,34]. Both mouse and rat are accepted in swimming exercise protocols, in this view, we used the rat model precisely by fact that rats TGI exhibit more similarities to humans, being more sensitive to the cholinergic transmission [34,35]. The animals rested for 48 h at the end of each week of exercise [36–38].

### Contractile reactivity measurement

Animals were killed by cervical dislocation followed by exsanguination. The ileum was immediately removed, cleaned of fat and connective tissue, immersed in physiological solution at room temperature and bubbled with carbogen mixture. To register the isotonic contractions, ileum segments (2-3 cm) were suspended by cotton yarn in organ bath (5 ml) and recorded on smoked drum through levers coupled to kymographs (DTF) under resting tension of 1.0 g at 37°C [39]. The organ baths were warmed by a thermostatic pump Polystat 12002 Cole-Palmer (Vernon Hills). The physiological solution used was Tyrode solution, whose pH was adjusted to 7.4 and the composition (in mM) was: NaCl (150.0), KCl (2.7), CaCl_2_ (1.8), MgCl_2_ (2.0), NaHCO_3_ (12.0), NaH_2_PO_4_ (0.4), D-glucose (5.5). After 30 min of stabilization period, an isotonic contraction was induced with 30 mM KCl to verify the functionality of the organ, 15 min after, two similar cumulative concentration-response curves to KCl (10^−3^ × 10^−1^ M) and CCh (10^−9^ × 10^−4^ M) were obtained. The contractile reactivity was assessed based on the values of the concentration of a substance that produces 50% of its maximal effect (EC_50_) and the maximum effect (*E*_max_) of contractile agents to the control and exercise groups. Tyrode's solution, KCl and CCh were diluted in freshly prepared distilled water time of the experiment.

### Lipid peroxidation assay

Lipid peroxidation in tissue was determined measuring the chromogenic product of 2-thiobarbituric acid (TBA) reaction with malondialdehyde (MDA), which is one of the products formed as a result of membrane lipid peroxidation [40]. Ileum segments of each animal were homogenized with KCl (1:1) and 250 μl of tissue homogenate were incubated at 37°C for 60 min. Then, the mixture was precipitated with 35% perchloric acid and centrifuged at 1207 ***g*** for 20 min at 4°C. The supernatant was transferred to Eppendorfs and 400 μl of 0.6% TBA were added and incubated at 95°C–100°C for 1 h. After cooling, the samples were read in spectrophotometer at a wavelength of 532 nm. The results were expressed in μmol/l per gram of dry tissue.

### Histological analysis

Samples of ileum were fixed in 10% formaldehyde solution, subjected to paraffin embedding standard histological procedures. This procedure included the following steps: (1) dehydration of the tissue at increasing alcohol series, 70% for 24 h, 80, 96 and 100% (third bath) for 1 h each; (2) diaphanization or bleaching in which the tissue was immersed in 100% xylene alcohol (1:1) for 1 h followed by two immersions in pure xylene for 1 h each; (3) impregnation in paraffin, wherein the sample was immersed in two baths of liquid paraffin (heated to 50°C) for 1 h each. The blocks obtained were cut to 5 μm thick in cross-section of the ileum using a rotary microtome. The sections were stained with Mayer's haematoxylin and eosin [41]. The slides were analysed with an optical microscope with an attached camera, where two cross-sections per animal were photographed and analysed. The second quadrant of the ileum circumference was analysed and measurements of the circular and longitudinal muscle layers were obtained in analyser program images [42].

### Statistical analysis

Data were expressed as mean ± S.E.M.. Cumulative concentration-response curves were fitted and EC_50_ values were obtained by non-linear regression [43]. Comparison of two groups was performed using the Student's *t*test and multiple comparisons by one-way ANOVA followed by Bonferroni's post-test. The differences were considered significant when *P*<0.05. All data were analysed using GraphPad Prism® software version 5.01 (GraphPad Software Inc.).

## RESULTS

### Contractile reactivity measurement

Cumulative concentration-response curves to KCl (*n*=5) were attenuated, with reduction on *E*_max_ value from 100% (SED) to 63.1±3.9; 48.8±3.8; 19.4±1.8 and 59.4±2.8% in the groups exercised by 2, 4, 6 and 8 weeks respectively. However, the EC_50_ values of the exercised groups (EC_50_=1.5±0.1; 1.9±0.4; 2.6±0.3 and 2.4±0.2×10^−2^ M respectively) showed no statistical difference when compared with SED group (EC_50_=1.7±0.1×10^−2^ M; Figure 1, Table 1). Similarly, cumulative concentration-response curves to CCh (*n*=5) were attenuated due to the exercise, with reduction in *E*_max_ value from 100% (SED) to 74.1±5.4; 75.9±5.2; 62.9±4.6 and 89.7±3.4% in the groups exercised by 2, 4, 6 and 8 weeks respectively. In contrast, the EC_50_ values of the exercised groups (EC_50_=1.5±0.5; 1.3±0.2; 1.5±0.3×10^−6^ and 2.1±0.4×10^−7^ M respectively) have showed no statistical difference when compared with the SED group (EC_50_=1.0±0.1×10^−6^ M). However, differences in the values of EC_50_ between EX2 compared with EX8 and EX6 compared with EX8 groups were observed (Figure 2, Table 2).

**Figure 1:**
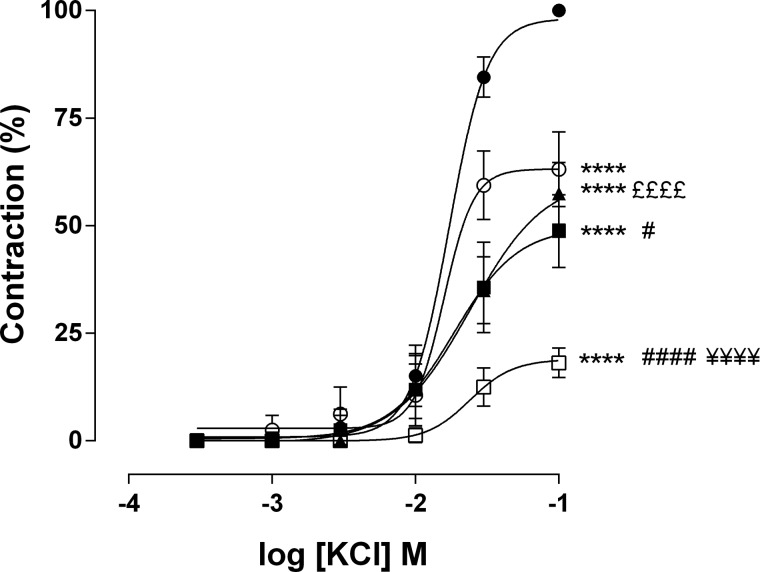
Cumulative concentration-response curves to KCl in the SED (cf%), EX2 (cb%), EX4 (aa%), EX6 (a1%) and EX8 groups (▲) on rat ileum The symbols and vertical bars represent the mean ± S.E.M. respectively (*n*=5). One-way ANOVA followed by Bonferroni's post-test. ^#^*P*<0.05 (EX4 compared with EX2), ^****^P<0.0001 (SED compared with EX2/EX4/EX6/EX8); ^####^ *P*<0.0001 (EX2 compared with EX6); ^†^*P*<0.0001 (EX4 compared with EX6); ^££££^*P*<0.0001 (EX6 compared with EX8).

**Table 1:** Values of EC_50_ (M) and *E*_max_ (%) of KCl in the SED, EX2, EX4, EX6 and EX8 groups on rat ileum

KCl (M)	EC_50_ (M)	*E*_max_ (%)
SED	1.7±0.1×10^−2^	100
EX2	1.5±0.1×10^−2^	63.1±3.9*
EX4	1.9±0.4×10^−2^	48.8±3.8^*†^
EX6	2.6±0.3×10^−2^	19.4±1.8^*‡§^
EX8	2.4±0.2×10^−2^	59.4±2.8^*║^

One-way ANOVA followed by Bonferroni's post-test (*n*=5). *P<0.0001 (SED compared with EX2/EX4/EX6/EX8); ^†^*P*<0.05 (EX2 compared with EX4); ^‡^*P*<0.0001 (EX2 compared with EX6); ^§^*P*<0.0001 (EX4 compared with EX6); ^║^*P*<0.0001 (EX6 compared with EX8).

**Figure 2:**
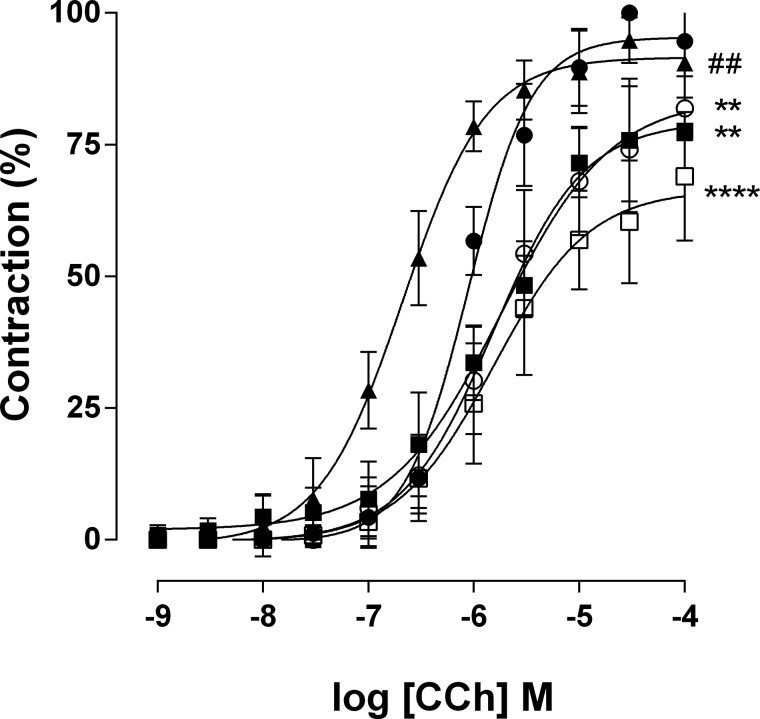
Cumulative concentration-response curves to CCh in the SED (cf%) and EX2 (cb%), EX4 (aa%), EX6 (a1%) and EX8 (▲) groups on rat ileum The symbols and vertical bars represent the mean ± S.E.M. respectively (*n*=5). One-way ANOVA followed by Bonferroni's post-test. **P<0.01 (SED compared with EX2/EX4); ^##^*P*<0.01 (EX6 compared with EX8); ^****^P<0.0001 (SED compared with EX6.).

**Table 2:** Values of EC_50_ (M) and *E*_max_ (%) of CCh in the SED, EX2, EX4, EX6 and EX8 groups on rat ileum

CCh (M)	EC_50_ (M)	*E*_max_ (%)
SED	1.0±0.1×10^−6^	100
EX2	1.5±0.5×10^−6^	74.1±5.4*
EX4	1.3±0.2 ×10^−6^	75.9±5.2*
EX6	1.5±0.3×10^−6^	62.9±4.6^†^
EX8	2.1±0.4×10^−7‡§^	89.7±3.4^║^

One-way ANOVA followed by Bonferroni's post-test (*n*=5). *P<0.05 (EX2 compared with EX8); ^†^*P*<0.05 (EX6 compared with EX8). ^‡^*P*<0.01 (SED compared with EX2/EX4); ^§^*P*<0.01 (EX6 compared with EX8); ^║^*P*<0.0001 (SED compared with EX6).

### Lipid peroxidation assay

The MDA concentration (*n*=5) in rat ileum was increased from 20.6±3.6 (SED) to 44.3±4.4 μmol/l/g of dry tissue (EX4), but did no differ on groups exercised by 2, 6 and 8 weeks (28.1±4.8; 20.0±3.6 and 17.2±3.6 μmol/l/g of dry tissue respectively) when compared with the control (Figure 3).

**Figure 3:**
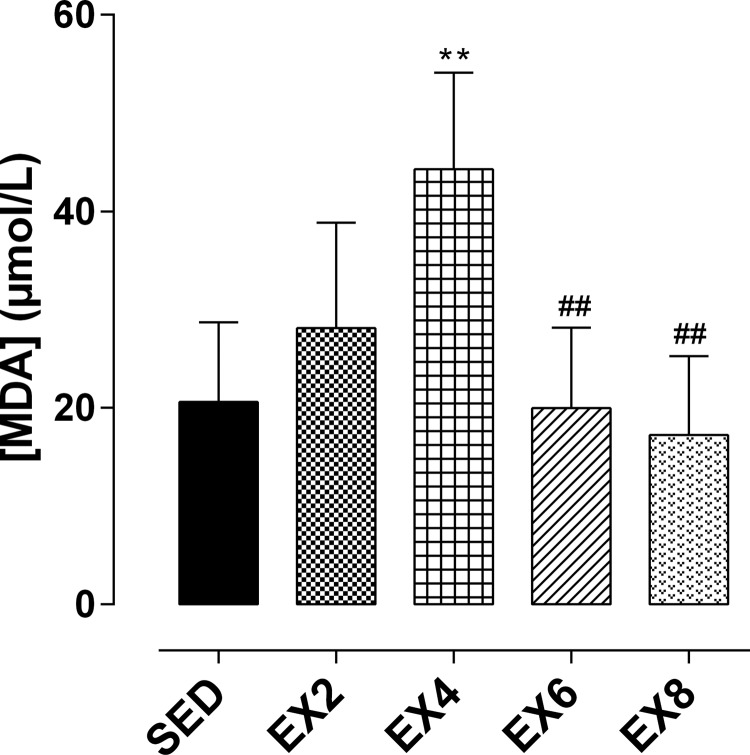
Concentration of MDA in the SED, EX2, EX4, EX6 and EX8 groups on rat ileum The columns and vertical bars represent the mean ± S.E.M. respectively (*n*=5). One-way ANOVA followed by Bonferroni's post-test. **P<0.01 (SED compared with EX4); ^##^*P*<0.01 (EX4 compared with EX4 and EX6 compared with EX8).

### Histologic analysis

The smooth circular muscle layer (*n*=5) was decreased from 50.9±0.3 (control) to 44.0±1.8, 43.5±1.3, 35.5±1.4 and 41.6±0.6 μm in groups exercised by 2, 4, 6 and 8 weeks respectively (Figures 4 and 5). However, longitudinal smooth muscle layer (*n*=5) was increased from 21.6±0.3 (control) to 31.8±1.0, 36.2±2.5, 29.6±1.8 and 30.8±1.3 μm in groups exercised by 2, 4, 6 and 8 weeks respectively (Figures 4 and 6).

**Figure 4:**
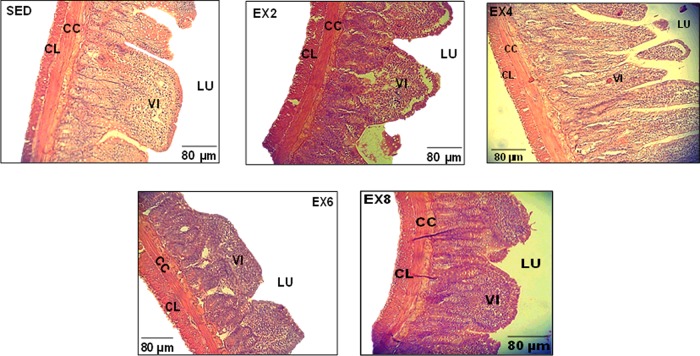
Histological section of the rat ileum from the SED, EX2, EX4, EX6 and EX8 groups Increased lens: 40×. Abbreviations: CL, circular layer; LL, longitudinal layer; LU, lumen, VI, villi.

**Figure 5:**
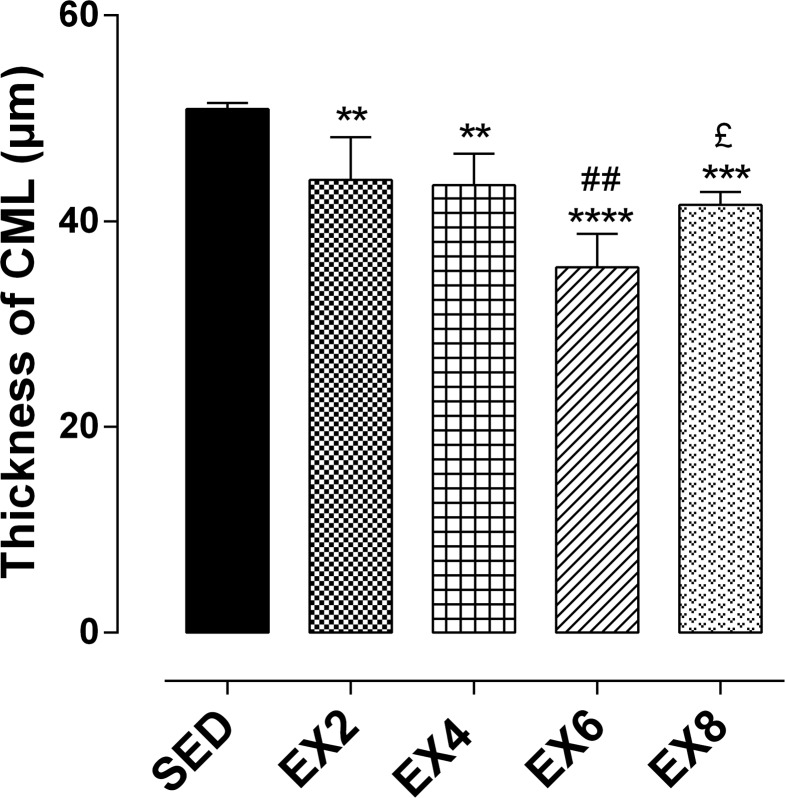
Circular muscle layer (CML) thickness of the SED, EX2, EX4, EX6 and EX8 groups The columns and vertical bars represent the mean ± S.E.M. respectively (*n*=5). One-way ANOVA followed by Bonferroni's post-test. ^£^*P*<0.05 (EX6 compared with EX8), **P<0.01 (SED compared with EX2/EX4); ^##^*P*<0.01 (EX2/EX4 compared with EX6); ***P<0.001 (SED compared with EX8); ^****^P<0.0001 (SED compared with EX6).

**Figure 6:**
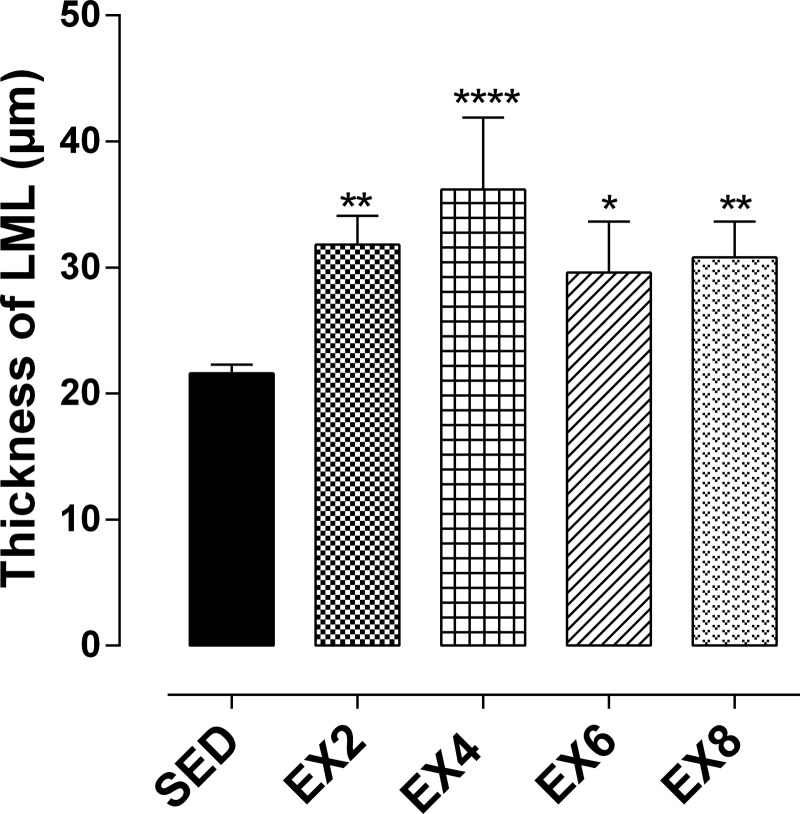
Longitudinal muscle layer (LML) thickness of the SED, EX2, EX4, EX6 and EX8 groups The columns and vertical bars represent the mean and S.E.M. respectively (*n*=5). One-way ANOVA followed by Bonferroni's post-test.); *P<0.05 (SED compared with EX6); **P<0.01 (SED compared with EX2/EX8); ^****^P<0.0001 (SED compared with EX4).

## DISCUSSION

In the present study, we investigated the influence of chronic aerobic swimming exercise on the contractile reactivity, oxidative stress and morphology on rat ileum, being shown that this type of exercise decreases the contractile response, the tissue lipid peroxidation and modify the thickness of intestinal smooth muscle layer.

Abnormalities on intestinal contractility represent one of the pathophysiological processes that characterize intestinal disorders, such as colic, diarrhoea and constipation [20]. Thus, physical exercise has become a growing practice in development, which has been considered an important therapeutic tool in the prevention and treatment of diseases affecting the GI tract [7].

Swimming exercise can be used to identify physiological, biochemical and molecular responses against the adjustments caused by chronic training [44–46] similar to found in experiments with human [27–30]. Regarding the GI tract, individuals who practice swimming exercise can present GI alterations [47]; however, the precise mechanism involved in these effects remains unclear.

Exercise taken in long-term is a stimulus for physiological adaptations. These adjustments are reflected by changes in contractile proteins, mitochondrial function, metabolic changes, intracellular signalling and transcriptional response [48]. In a study performed by Rosa et al. [49], it was observed that chronic aerobic treadmill exercise does not affect the contractile reactivity of old mice ileum. In addition, Lira et al. [42] showed that the same chronic exercise alters the ileum contraction reactivity of young mice, decreasing the response to acetylcholine. This effect was associated to changes in plasma concentration of hormones that regulate the function of GI tract. The authors analysed the influence of treadmill exercise on contractile reactivity of mouse ileum in exercise periods of 10, 25, 40 and 55 days and it was verified that the ileum contractile reactivity was increased from 10 days training, but it was decreased from 55 days, highlighting the importance of chronic evaluations. In fact, the time necessary to these adaptations occur varies depending on type and time of exercise [50]. Additionally, differences in physiological responses have been demonstrated in rats treadmill or swimming exercised, being observed that treadmill exercise is more effective in modulating peripheral serotonergic system, whereas swimming has greater influence on sympathetic nervous system [51], increasing the adrenaline release [52].

Therefore, since there are differences in physiological changes obtained in various modalities of exercise, as well as studies show that treadmill exercise reduces mouse ileum contractile reactivity, it was decided to investigate whether chronic aerobic swimming exercise could also alter rat ileum contractile reactivity.

It was verified that the exercise attenuated the cumulative concentration-response curves to KCl, an electromechanical contractile agent [53], decreasing its efficacy without changing the contractile potency (Figure 1). Similarly, the exercise attenuated the cumulative concentration-response curves to CCh, which acts by a pharmacomechanical coupling [54], decreasing its efficacy with increasing on the EX8 potency, which can be associated to the adaptation process to the exercise (Figure 2). In addition, it was verified that exercise altered the rat ileum contractile reactivity from second to sixth week, promoting a decrease on contractile responsiveness to both contractile agents. In contrast, in the eighth week of exercise (EX8), the cumulative curve to KCl was increased compared with the exercised group for 6 weeks, do no differing to groups exercised for 2 and 4 weeks, but not completely restored to the control group, whereas the cumulative curve to CCh for this group was similar to that achieved in the SED group, representing a complete restauration of the contractile response to this agonist (Figures 1 and 2). Thus, the rat ileum contractile reactivity undergoes adaptive responses from the first few weeks of exercise and attains a stabilization period on the eighth week. This data set indicate that chronic aerobic swimming exercise decreases rat ileum responsiveness front both contractile agents, possibly due to an increase on the noradrenaline release during the swimming exercise [51]. Furthermore, we cannot discharge that, perhaps, the swimming exercise alters the cholinergic transmission and the resting membrane potential of intestinal smooth muscle cell, which could be the responsible for the contractile response attenuation.

The physiological changes produced on chronic exercises promote increase in antioxidants that remove FRs and stabilize the production of reactive species [15]. Silva et al. [55] demonstrated a decrease in MDA levels in mice skeletal muscle after 8 weeks of treadmill exercise. However, in a study developed by Rocha et al. [56], it was observed that a period of 8 weeks of swimming exercise did not decreased the levels of lipid peroxidation in rat aorta. In contrast, it was observed an increased expression of the antioxidant superoxide dismutase (SOD) enzyme, but without changing the concentration of catalase (CAT), leading to an imbalance in the SOD/CAT. It is related that this imbalance in SOD/CAT can explain the increase in tissue lipid peroxidation, leading to oxidative damage, promoting changes in their structure and function. However, it is reported that minor damage on tissue structure is needed to promote adaptation, functioning as a cellular stimulus.

To verify whether the swimming exercise reduces oxidative damage on rat ileum, it was investigated the production of MDA, indicating possible alterations in the lipid matrix and cell membranes resulting from the indirect effect of ROS production [13]. We showed that chronic aerobic swimming exercise increases lipid peroxidation after 4 weeks of exercise and decreases after 6 weeks (Figure 3). These data support the hypothesis that chronic aerobic exercise increases lipid peroxidation in this tissue after 4 weeks of exercise and it served as a cellular stimulus for adaptation to exercise and to stimulate the cellular production of antioxidant to remove FRs and reduce oxidative damage promoted by the exercise itself.

Since there was an increase in lipid peroxidation, indicating an increase in FRs production on rat ileum during 4 weeks of exercise and knowing that the increase in FRs causes oxidative stress with potential damage to tissues and organs [15,57,58], we hypothesized that this increase would be changing the architecture of intestinal smooth muscle. To assess this hypothesis, we measured the muscle layer thickness through histological sections of rat ileum and we verified a decrease on the circular muscle layer thickness (Figures 4 and 5) and an increase on the longitudinal muscle layer thickness (Figures 4 and 6) in all exercised groups.

Given these results, we concluded that lipid peroxidation is not related to alterations in muscle layer thickness, since there was an increase in lipid peroxidation in the group exercised for 4 weeks, whereas the alterations of the smooth muscle layers occurred in all the exercised groups. Thus, chronic aerobic swimming exercise alters the structure of the tissue, probably not due to the increase in reactive species production; however, other mechanism seems to be related to this change in the structure of smooth muscle acting as a stimulus to the organ adaptation.

The contractile reactivity of circular and longitudinal smooth muscle layers of rat ileum is affected differently by the hypertrophic process. Hypertrophy of the circular layer shows an increase in contractile efficacy, whereas hypertrophy of the longitudinal layer exhibits a greater sensitivity to relaxing factors, leading to decreased contraction [59,60]. These data corroborate our results that showed a decrease in circular and increase in longitudinal layer, with reduction in contractile reactivity.

It is well known that the two most common symptoms in patients affected by chronic diseases, such as Crohn's Disease, are diarrhoea and constipation [61]. It has been suggested that physical exercise has beneficial effects on the GI tract, mainly due to decreased GI blood flow, neuroimmuno-endocrine alternations and increased GI motility [7]. Additionally, some studies have shown that physical activity can accelerate orocecal transit time in different populations and improve symptoms of constipation in irritable bowel disease patients [62].

Therefore, our results provide some initial evidences that the swimming physic exercise, in animal model, modifies the intestinal motility due to mechanisms involving the cholinergic transmission as well as affecting the resting membrane potential. To what extent these findings can be extrapolated to human to explain the reducing in intestinal motility is a point that need more research.

## Abbreviations

CCh: carbamylcholine hydrochloride
CML: circular muscle layer
EX2/4/6/8: exercised for 2/4/6/8 weeks
FR: free radical
GI: gastrointestinal
LML: longitudinal muscle layer
MDA: malondialdehyde
RNS: reactive nitrogen species
ROS: reactive oxygen species
SED: sedentary
SOD: superoxide dismutase
TBA: 2-thiobarbituric acid
